# Antennal Transcriptome Analysis of the Chemosensory Gene Families From Trichoptera and Basal Lepidoptera

**DOI:** 10.3389/fphys.2018.01365

**Published:** 2018-09-27

**Authors:** Jothi Kumar Yuvaraj, Martin N. Andersson, Dan-Dan Zhang, Christer Löfstedt

**Affiliations:** Department of Biology, Lund University, Lund, Sweden

**Keywords:** odorant receptor, gustatory receptor, ionotropic receptor, odorant binding protein, chemosensory protein, pheromone, sensory neuron membrane protein

## Abstract

The chemosensory gene families of insects encode proteins that are crucial for host location, mate finding, oviposition, and avoidance behaviors. The insect peripheral chemosensory system comprises odorant receptors (ORs), gustatory receptors (GRs), ionotropic receptors (IRs), odorant binding proteins (OBPs), chemosensory proteins (CSPs), and sensory neuron membrane proteins (SNMPs). These protein families have been identified from a large number of insect species, however, they still remain unidentified from several taxa that could provide important clues to their evolution. These taxa include older lepidopteran lineages and the sister order of Lepidoptera, Trichoptera (caddisflies). Studies of these insects should improve evolutionary analyses of insect chemoreception, and in particular shed light on the origin of certain lepidopteran protein subfamilies. These include the pheromone receptors (PRs) in the “PR clade”, the pheromone binding proteins (PBPs), general odorant binding proteins (GOBPs), and certain presumably Lepidoptera-specific IR subfamilies. Hence, we analyzed antennal transcriptomes from *Rhyacophila nubila* (Trichoptera), *Eriocrania semipurpurella*, and *Lampronia capitella* (representing two old lepidopteran lineages). We report 37 ORs, 17 IRs, 9 GRs, 30 OBPs, 7 CSPs, and 2 SNMPs in *R. nubila*; 37 ORs, 17 IRs, 3 GRs, 23 OBPs, 14 CSPs, and 2 SNMPs in *E. semipurpurella*; and 53 ORs, 20 IRs, 5 GRs, 29 OBPs, 17 CSPs, and 3 SNMPs in *L. capitella*. We identified IR members of the “Lepidoptera-specific” subfamilies IR1 and IR87a also in *R. nubila*, demonstrating that these IRs also occur in Trichoptera. Members of the GOBP subfamily were only found in the two lepidopterans. ORs grouping within the PR clade, as well as PBPs, were only found in *L. capitella*, a species that in contrast to *R. nubila* and *E. semipurpurella* uses a so-called Type I pheromone similar to the pheromones of most species of the derived Lepidoptera (Ditrysia). Thus, in addition to providing increased coverage for evolutionary analyses of chemoreception in insects, our findings suggest that certain subfamilies of chemosensory genes have evolved in parallel with the transition of sex pheromone types in Lepidoptera. In addition, other chemoreceptor subfamilies show a broader taxonomic occurrence than hitherto acknowledged.

## Introduction

Chemoreception is of utmost importance for the survival and reproduction of insects. The insect antenna is the main olfactory appendage, and it is covered with numerous sensory structures, called sensilla (Keil, 1999; Yuvaraj et al., 2018a). The olfactory sensilla contain the dendrites of olfactory sensory neurons (OSNs), which house chemoreceptors from three divergent multi-gene families, i.e., the odorant receptor (OR) (Clyne et al., 1999; Gao and Chess, 1999; Vosshall et al., 1999), gustatory receptor (GR) (Kwon et al., 2007), and ionotropic receptor (IR) (Benton et al., 2009) families. The receptors and additional families of non-receptor proteins involved in chemosensation (Leal, 2013) have now been identified in many species (Montagné et al., 2015), providing a basis for subsequent evolutionary, structural, and functional studies of these molecular cornerstones of olfaction and taste. However, the chemosensory gene families have, to our knowledge, not yet been identified from certain taxa which could provide important clues to their evolution and origin, including the Trichoptera (caddisflies, the sister group of Lepidoptera) and older lineages of Lepidoptera comprising the so-called non-ditrysian moths (Löfstedt et al., 2016).

Insect ORs are seven-transmembrane proteins with an intracellular N-terminus and extracellular C-terminus, which is opposite to the topology of the G protein-coupled ORs of vertebrates. No homology is shared between these two groups of ORs (Clyne et al., 1999; Benton et al., 2006; Wistrand et al., 2006). In insects, each ligand-binding OR forms a heterotetrameric complex (Butterwick et al., 2018) with an evolutionary conserved odorant receptor co-receptor (Orco) (Vosshall and Hansson, 2011; Stengl and Funk, 2013; Corcoran et al., 2018). Orco is necessary for odor responses (Sato et al., 2008; Wicher et al., 2008), and also important for the ORs to localize in the cell membrane of OSN dendrites (Larsson et al., 2004; Benton et al., 2006; German et al., 2013). With few exceptions, (e.g., Dobritsa et al., 2003; Koutroumpa et al., 2014; Karner et al., 2015) each OSN expresses only one odorant-binding OR, which to a large extent determines the response profile of the OSN. The OR repertoire is highly divergent across insects, both in terms of sequence variation and the total number of ORs encoded by different genomes (Andersson et al., 2015; Montagné et al., 2015). This diversity is thought to at least partly depend on the specialization to different ecological niches (Nei et al., 2008; Hansson and Stensmyr, 2011; Andersson et al., 2015). The GRs belong to the same superfamily as the ORs (Robertson et al., 2003). The GRs are mainly expressed in taste organs and are involved in contact chemoreception (Vosshall and Stocker, 2007), but this gene family also encodes conserved receptors for carbon dioxide, often expressed in the insect antennae (Kwon et al., 2007; Robertson and Kent, 2009).

IRs are related to ionotropic glutamate receptors (iGluRs) that represent a conserved family of ligand-gated ion channels that mediate neuronal communication at synapses in both vertebrates and invertebrates. However, the IRs have atypical binding domains and are involved in sensing the external environment (Benton et al., 2009). The class of ‘antennal’ IRs comprises receptors involved in olfaction (Benton et al., 2009; Croset et al., 2010; Rytz et al., 2013), humidity (Enjin et al., 2016; Frank et al., 2017), temperature (Chen et al., 2015), and salt sensing (Zhang et al., 2013). These IRs are conserved across insect orders (Croset et al., 2010; Rytz et al., 2013). On the other hand, the ‘divergent’ IRs are highly variable across species, and members of this class have been assigned a putative role in taste (Croset et al., 2010). A third, phylogenetically distinct, group of IRs occurs in moths and butterflies, and was recently proposed to be Lepidoptera-specific (Liu et al., 2018). In contrast to the ORs, IRs are expressed in a combinatorial fashion in OSNs, and they are in *Drosophila* tuned to different sets of odorants, notably acids, aromatics, and nitrogen-containing compounds (Abuin et al., 2011). The IRs represent a more ancestral receptor family than the OR family as evidenced by their presence throughout protostome lineages (Croset et al., 2010; Eyun et al., 2017).

Proteins encoded by additional gene families are also important for olfaction. The sensory neuron membrane proteins (SNMPs) are expressed in certain OR-expressing OSNs. SNMPs are integral membrane proteins, related to scavenger proteins, and belonging to the CD36 family. Two representatives of SNMPs (SNMP1 and SNMP2) are found in insects (Nichols and Vogt, 2008), although some insect genomes encode multiple numbers of each group with six putative SNMP1 members being the highest number reported so far (Andersson et al., 2014, 2016). SNMP1 has an important role in pheromone detection in *Drosophila* and moths (Benton et al., 2007; Li et al., 2014; Pregitzer et al., 2014; Gomez-Diaz et al., 2016). In addition, the protein-rich lymph inside sensilla contains odorant binding proteins (OBPs) involved in the binding and solubilization of odorants in the lymph (Große-Wilde et al., 2006; Leal, 2013). OBPs are small (typically 135–220 amino acids) hydrophilic proteins with conserved cysteine residues forming disulfide bridges (Vogt, 2003; Sánchez-Gracia et al., 2009). Two specialized monophyletic subfamilies of OBPs, the pheromone binding proteins (PBPs) and general odorant binding proteins (GOBPs), appear to be conserved in most species of the higher Lepidoptera (Ditrysia) (Vogt et al., 2015). However, it remains unknown if they are present also in older moth lineages or in caddisflies that use a different chemical type of sex pheromone (Löfstedt et al., 2016). Similar to the OBPs, the insect chemosensory proteins (CSPs) are thought to bind hydrophobic molecules. These proteins are also small (generally around 130 amino acids), and characterized by four conserved cysteine residues forming two disulfide bridges. However, CSPs are also expressed in a variety of non-sensory tissues, and their importance in olfaction and taste remain unclear (Pelosi et al., 2006; Sánchez-Gracia et al., 2009).

**FIGURE 1:**
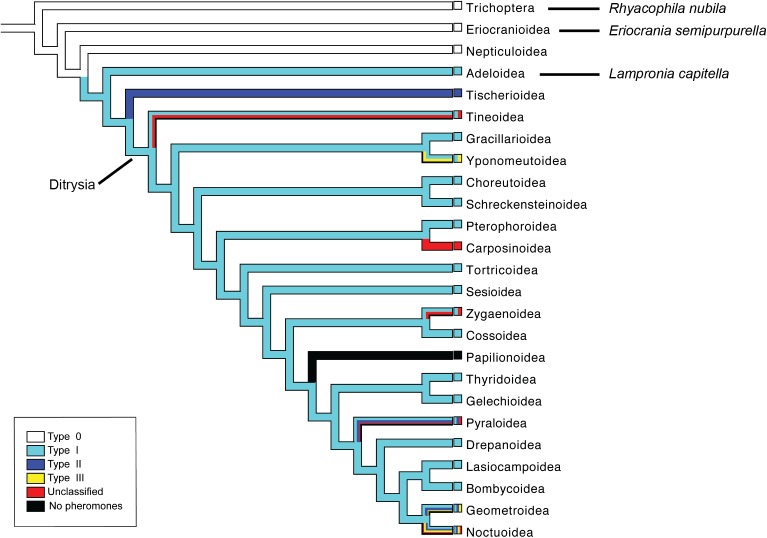
Phylogeny of major lepidopteran superfamilies and the sister order Trichoptera with the suggested evolution of different sex pheromone types indicated by different colors on branches (adapted from Löfstedt et al., 2016). Except for the Papilionoidea, only taxa with identified female-produced sex pheromones or sex attractants are included. The figure is modified from Yuvaraj et al. (2017), with permission from the publisher (link to article: https://academic.oup.com/mbe/article/34/11/2733/4060563).

The order Trichoptera contains species with aquatic immature stages, and together with the Lepidoptera, they form the suborder Amphiesmenoptera (Kjer et al., 2001; Kjer et al., 2002). Among the Lepidoptera, the early-diverging families Eriocraniidae and Prodoxidae belong to the non-ditrysian group of moths (**Figure 1**). The leaf miner moth, *Eriocrania semipurpurella* (Eriocraniidae) uses Type 0 sex pheromone compounds (short chain secondary alcohols or ketones) similar to the pheromones used by species in the sister group Trichoptera (**Figure 1**; Löfstedt et al., 2016). The currant shoot borer, *Lampronia capitella* (Prodoxidae) uses Type I pheromone compounds (C_10_–C_18_ acetates, alcohols and aldehydes) for sex communication, which is the most common type of sex pheromone in ditrysian moths (Löfstedt et al., 2016). Based on currently available pheromone data within the Lepidoptera, Adeloidea to which Prodoxidae belongs, is the earliest diverging branch in the phylogeny with species using Type I pheromone compounds (**Figure 1**; Löfstedt et al., 2016).

During the last decades, the chemosensory gene families have been extensively studied in ditrysian Lepidoptera and many other groups of insects (e.g., Krieger et al., 2002; Grosse-Wilde et al., 2011; Bengtsson et al., 2012; Andersson et al., 2013, 2014; Corcoran et al., 2015; Walker et al., 2016). On the other hand, the early-diverging lineages of Lepidoptera and its sister group Trichoptera are poorly studied in terms of their chemosensory genes, probably due to the fact that most of these species are not known to be pests of agricultural crops. Among these taxa, there has been a transition in pheromone types from Type 0 to Type I compounds, seemingly representing a major evolutionary transition in terms of chemical communication (Löfstedt et al., 2016). The ORs of *E. semipurpurella* and *L. capitella* were reported in our previous functional characterization studies (Yuvaraj et al., 2017, 2018b), but not the other chemosensory gene families. We aim to find out whether the evolution of different pheromone types is associated with complementary changes in the periphery of the pheromone detection system. For example, changes in chemosensory genes may be associated with the transition to Type I pheromones. Hence, we analyzed antennal transcriptomes of *L. capitella* that belongs to the earliest diverging moth lineage that uses Type I sex pheromones, *E. semipurpurella* belonging to an even older moth lineage using Type 0 sex pheromones, and *Rhyacophila nubila* that belongs to the Trichoptera, which also use Type 0 sex pheromones. We report the antennally expressed ORs, IRs, GRs, OBPs, CSPs, and SNMPs in these three species. We also estimate their expression levels based on RNAseq data, and analyze their taxonomic occurrence and evolutionary relationships with the corresponding proteins in moths of the Ditrysia. We reveal that certain subfamilies of chemosensory genes only appear in antennal transcriptomes of moths using Type I sex pheromones, whereas other subfamilies occur more broadly than previously realized. Hence, our results contribute to a better understanding of the evolution of semiochemical communication systems within the superorder Amphiesmenoptera.

## Materials and Methods

### Insects

Pupae of *R. nubila* were collected from a river stream outside Sjöbo in southernmost Sweden (55°41′13.2″N 13°21′24.6″E, 88.06 m alt.), and kept at 14–16°C and 16:8 h light:dark cycle with external aeration in an aquarium for adults to emerge. Adult males of *E. semipupurella* were collected from a birch forest near Skrylle in southernmost Sweden (55°38′51.0″N 13°41′28.1″E, 39.53 m alt.) using traps baited with sex pheromone (Kozlov et al., 1996; Yuvaraj et al., 2017). Adults of male and female *L. capitella* were collected by hand from a black currant plantation near Roskilde, Denmark (55°36′26.8″N 11°58′35.2″E, 14.54 m alt.).

### RNA Extraction and First-Strand cDNA Synthesis

Antennae were removed from the males and females under a stereo microscope (Olympus SZ Series Zoom, Olympus, Tokyo, Japan) and stored at -80°C until RNA isolation. Total RNA from pools of antennae (50 pairs from each sex of *R. nubila*, 100 pairs from male *E. semipurpurella*, and 100 pairs from each sex of *L. capitella*) was extracted using the Trizol protocol (Thermo Fisher Scientific, Carlsbad, CA, United States), and subsequently purified using an RNA Purification Kit (Invitrogen, Carlsbad, CA, United States) according to the manufacturer’s instructions. The concentration and quality of the RNA were analyzed using a Nanodrop2000 (Thermo Scientific, Saveen Werner, Malmö, Sweden). First-strand cDNA was synthesized from 1 μg of DNAse-treated total RNA using the ThermoScript RT-PCR system for First-Strand cDNA Synthesis (Thermo Fisher Scientific) following the manufacturer’s instructions, except that both oligo-dT primers and random hexamers (1 μL of each) were used in the 20 μL reaction mixtures. The first-strand cDNA library was then used for cloning of chemosensory genes (see section “PCR Confirmation and RACE-PCR Amplification”).

### Sequencing, Assembly and Annotation

The cDNA libraries were sequenced paired-end (100 bp) using an Illumina HiSeq 2000 platform (Illumina, San Diego, CA, United States) at the Beijing Genomics Institute (BGI Hong Kong Co. Ltd.,) and RNAseq libraries were constructed using Illumina’s standard protocols. Adaptor sequences were removed and low quality reads trimmed using Trimmomatic^1^. *De novo* transcriptome assembly of the cleaned data was then performed using the short reads assembly program Trinity (default parameters, version 20121005, Grabherr et al., 2011), and the assembled reads were clustered by TGICL (Pertea et al., 2003). Male and female derived reads were assembled both separately and combined. Primarily the combined assemblies were used for the annotation of chemosensory genes. However, on a few occasions the open reading frames of certain chemosensory genes were longer in the sex-specific assemblies, and were in these cases included in the final dataset. The completeness of each of the assembled transcriptomes (sexes combined for *R. nubila* and *L. capitella*) was assessed using the Benchmarking Universal Single-Copy Orthologs (BUSCOv3) tool performed against the Insecta odb9 dataset that includes 1658 reference genes^2^ (Waterhouse et al., 2017).

Annotations of chemosensory genes were performed as previously described (Yuvaraj et al., 2017). Briefly, all assembled transcripts were initially included in blast searches against the pooled database of non-redundant (nr) proteins at NCBI (National Center for Biotechnology Information), using an *e*-value cut-off at 1e-5. Transcripts with hits for putative chemosensory genes were identified from this blast search, open reading frames (ORFs) identified, and then verified by additional BLASTp searches against the nr database. Also, the identified chemosensory genes from the three species were used as queries in additional tBLASTn searches (*e*-value cut-off < 1e-1) against the transcriptomes of all three species to ensure that all chemosensory genes were identified. Searches against the Pfam web-tool from EMBL-EBI^3^ and transmembrane predictions using TMHMM server version 2.0^4^, were undertaken to further support the annotations. Apart from the functionally tested ORs in *E. semipurpurella* and *L. capitella* (Yuvaraj et al., 2017, 2018b), the sequences of ORs and OBPs were numbered in the order they were found in each transcriptome. No OR was given the number 2, to avoid confusion with previously reported lepidopteran Orco proteins. Two pairs of OBPs in *R. nubila* showed >75% identity, and were therefore given the same number (OBP18 or 26), but with an “a” or “b” suffix. SNMPs, IRs, and the OBP subfamilies PBPs and GOBPs were named according to sequence homology with other previously identified lepidopteran proteins. Similarly, putative GRs for carbon dioxide were named GR1-3 (Robertson and Kent, 2009), and putative sugar receptors were named according to sequence homology with such receptors in other moth species. GRs that were not annotated as putative carbon dioxide or sugar receptors were labeled consecutively from number 11. Finally, the CSPs were numbered consecutively based on their tree groupings. Transcript sequences encoding putative chemosensory genes with >99% amino acid identity were regarded as alleles or assembly isoforms and only one copy was included. We use the prefix Rnub for the chemosensory genes of *R. nubila*, Esem for *E. semipurpurella*, and Lcap for *L. capitella*.

The expression levels of transcripts were estimated using the FPKM method (fragments per kb transcript per million mapped reads). The expression of chemosensory genes was regarded as sex-biased if the FPKM values differed by >3-fold between the sexes. This more stringent cut-off compared to the standard twofold change was used due to lack of biological replication. Only genes that had FPKM values above 2 in at least one of the sexes were included in the analysis. The sequences, length details, and FPKM values of all identified chemosensory genes and proteins are presented in **Supplementary Data Sheets S1**–**S3**, for *R. nubila*, *E. semipurpurella*, and *L. capitella*, respectively.

### PCR Confirmation and RACE-PCR Amplification

To confirm the sequence of some transcripts encoding *R. nubila* ORs and *L. capitella* IRs (**Supplementary Data Sheet S3**), PCR amplification from cDNA, followed by cloning and Sanger sequencing were performed. Full length or partial genes were amplified using gene specific primers (oligonucleotide primer sequences are reported in **Supplementary Table S1**) and Platinum^®^ Pfu polymerase (Thermo Fisher Scientific), and adenosine residues were added to the ends of the PCR products using GoTaq^®^ Green Master mix (Thermo Fisher Scientific). The PCR products were resolved on 0.7% TAE agarose gels and bands of predicted length were cut and purified using the Wizard^®^ SV Gel and PCR clean-up system (Promega). The purified PCR products were transformed into the pTZ57R/T vector and colonies were tested for successful transformation. Positive colonies were grown in LB media (containing ampicillin) overnight, and plasmids were extracted using the GeneJET plasmid miniprep kit (Thermo Fisher Scientific). Sequencing PCR was performed using vector-specific primers and BigDye^®^ Terminator v1.1 Cycle Sequencing Kit (Thermo Fisher Scientific) following the manufacturer’s protocol. The plasmids were then Sanger sequenced using a capillary 3130xL Genetic Analyzer (Thermo Fisher Scientific) at the Department of Biology sequencing facility (Lund University, Lund, Sweden).

Assembled transcripts did not always encode full-length proteins of chemosensory genes, causing miss-alignments that prevented proper phylogenetic analyses of the ORs in particular. Hence, 5′ and 3′ RACE-PCR (50 μl reactions) was carried out for some of the short OR transcripts in *R. nubila* (**Supplementary Data Sheet S1** and **Supplementary Table S1**) to obtain full length sequences, using the SMARTer RACE cDNA Amplification Kit (Clontech, Mountain View, CA, United States) according to the manufacturer’s instructions. The following program was used: 5 cycles of 94°C for 30 s, 72°C for 3 min; 5 cycles of 30 s at 94°C, 30 s at 70°C, 3 min at 72°C; 20 cycles of 30 s at 94°C, 30 s at 68°C, 3 min at 72°C; and a final extension of 7 min at 72°C. Further cloning and sequencing was performed as described above.

### Phylogenetic Analyses

The amino acid sequences of predicted ORs, IRs, SNMPs, OBPs, and CSPs from *R. nubila*, *E. semipurpurella*, and *L. capitella* were aligned together with proteins from *Manduca sexta*, *Plutella xylostella* and *Epiphyas postvittana* using the MAFFT sequence alignment plugin in Geneious R7 software (Biomatters^5^). To improve the robustness of the phylogenetic analysis, miss-aligned sequences, OR sequences below 200 amino acids, and IR sequences below 100 amino acids were not included (except the 96-amino acid fragment of IR60a from *L. capitella*, which aligned well). The OR tree was rooted with the lineage of conserved Orco proteins, and the IR tree with the IR8a and IR25a subfamilies. To ensure correct naming of IRs, *Drosophila melanogaster* IR sequences were also included in the IR tree. A non-SNMP member of CD36 family (Croquemort, Dmelcrq-A) was used to root the SNMP tree. Maximum-likelihood phylogenetic trees were constructed with RAxML8 (Stamatakis, 2014), and branch support was obtained using 500 bootstrap replicates. The trees were visualized and color coded in FigTree V 1.4.2^6^.

## Results

### Assemblies

The Illumina sequencing of the *R. nubila* antennal samples yielded a total of 110 million reads from each sex. The reads from both sexes combined were assembled into 53,067 transcripts, with a mean length of 1005 bp and an N_50_ value of 1991 bp. In total, 65 million reads from the male *E. semipurpurella* antennal sample were assembled into 68,151 transcripts with a mean length of 818 bp and *N*_50_-value of 1,761. The antennal samples of *L. capitella* yielded 110 million reads from each sex. The reads from both sexes combined were assembled into 60,437 transcripts, with a mean length of 1022 bp and an *N*_50_ value of 2069 bp. The raw sequenced reads have been deposited in the Sequence Read Archive (SRA) database at NCBI under the Bioproject accession numbers: SRR7459244 (*R. nubila*), SRR5328787 (*E. semipurpurella*), and SRR6679363 (*L. capitella*). The transcriptome assemblies have been deposited in the Transcriptome Shotgun Assembly database at DDBJ/EMBL/GenBank under the accessions: GGRG00000000 (*R. nubila*), GFQP00000000 (*E. semipurpurella*), and GGRH00000000 (*L. capitella*). The versions described in this paper are the first versions: GGRG01000000 (*R. nubila*), GFQP01000000 (*E. semipurpurella*), and GGRH01000000 (*L. capitella*). BUSCO analysis using the Insecta odb9 dataset with 1658 reference genes revealed that the completeness of the transcriptomes was high, i.e., 91, 86, and 95%, for *R. nubila* (sexes combined), *E. semipurpurella* (male only), and *L. capitella* (sexes combined), respectively (for additional details, see **Supplementary Table S2**).

### Receptor Gene Families

#### Odorant Receptors

In previous studies reporting the functional characterization of sex pheromone receptors, we identified 37 ORs in *E. semipurpurella* (Yuvaraj et al., 2017) and 53 ORs in *L. capitella* (Yuvaraj et al., 2018b), including the co-receptor Orco. Here, we report 37 ORs from *R. nubila*, including Orco (**Table 1** and **Supplementary Data Sheet S1**). For *R. nubila*, two partial transcripts encoding ORs were extended to full-length using RACE-PCR (RnubOR5 and 8). The full-length sequences of nine additional RnubOR transcripts were confirmed from cDNA. Sequences of the cloned and RACE-PCR extended OR genes from the three studied species have been deposited in GenBank (see **Supplementary Data Sheet S4** for accession numbers).

**Table 1:** Number of genes identified for each chemosensory gene family in *Rhyacophila nubila, Eriocrania semipurpurella*, and *Lampronia capitella*.

	ORs	GRs	IRs	OBPs	CSPs	SNMPs
*Rhyacophila nubila*	37	9	17	30	7	2
*Eriocrania semipurpurella*	37	3	17	23	14	2
*Lampronia capitella*	53	5	20	29	17	3


*OR, odorant receptor; GR, gustatory receptor; IR, ionotropic receptor; OBP, odorant binding protein; CSP, chemosensory protein; SNMP, sensory neuron membrane protein.*

In total, 25 of the transcripts encoding RnubORs were regarded as full-length with more than 400 amino acids (**Supplementary Data Sheet S1**). Two of the longer partial OR fragments (OR24, and OR29) contained between 300 and 400 amino acids, but lacked the N- or C-terminus. Length-details of the OR-encoding transcripts in *E. semipurpurella* and *L. capitella* have been reported previously (Yuvaraj et al., 2017, 2018b), but in brief, 24 ORs are full length proteins in *E. semipurpurella*, and 37 ORs in *L. capitella* (**Supplementary Data Sheets S2**, **S3**).

**FIGURE 2:**
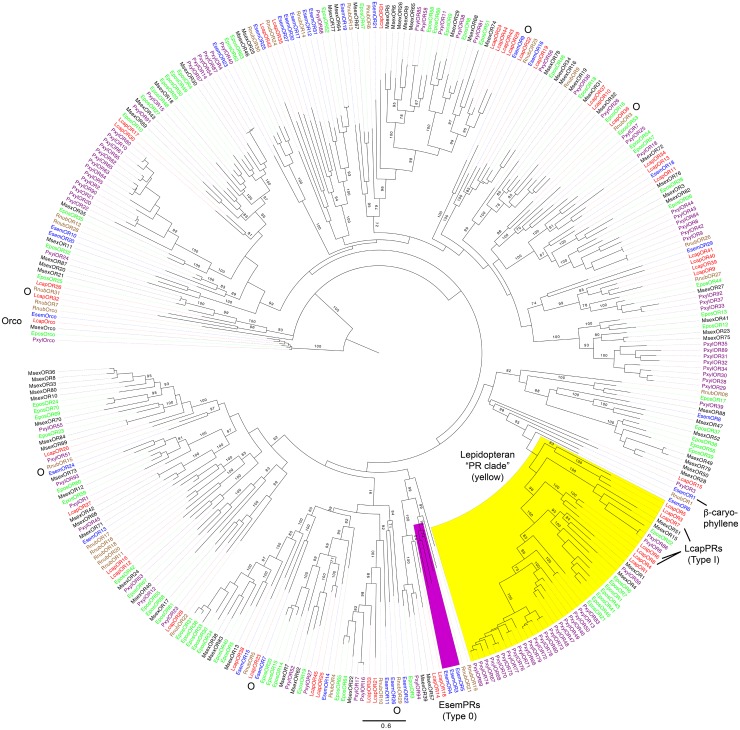
Maximum-likelihood phylogram of odorant receptors (ORs), rooted with the lineage of conserved Orco proteins. Included are sequences from *Rhyacophila nubila* (brown), *Lampronia capitella* (red), *Eriocrania semipurpurella* (blue), *Epiphyas postvittana* (green), *Manduca sexta* (black), and *Plutella xylostella* (purple). Highlighted clades: the lepidopteran ‘pheromone receptor (PR) clade’ in yellow according to Yuvaraj et al. (2018b), and the *E. semipurpurella* Type 0 pheromone receptor lineage in purple (Koenig et al., 2015; Yuvaraj et al., 2017, 2018b). The LcapPRs within the PR clade and the β-caryophyllene response of EsemOR1 are also highlighted. Bootstrap support values (500 replicates) are reported on major branches if larger than 70%. Putative simple (one-to-one) orthologous relationship with bootstrap support >70% are indicated with the letter “O”. Sources of included OR sequences, along with their accession numbers (when applicable), are reported in **Supplementary Data Sheet S4**.

Phylogenetic analysis of the *R. nubila, E. semipurpurella*, and *L. capitella* OR sequences was performed together with OR datasets from *M. sexta, E. postvittana*, and *P. xylostella*. As expected, the conserved Orco proteins from all species clustered together in a clade that was used to root the tree (**Figure 2**). No ORs from *R. nubila* or *E. semipurpurella* grouped within the recently extended lepidopteran pheromone receptor (PR) clade (**Figure 2**; Koenig et al., 2015; Yuvaraj et al., 2018b). In contrast, *L. capitella* has seven ORs that form two subfamilies within the PR clade (LcapORs 1, 4, 6, 8, and LcapORs 3, 5, 7, respectively), of which LcapORs 6–8 respond to Type I pheromone compounds (Yuvaraj et al., 2018b). Additionally, our phylogenetic analysis suggests that one RnubOR (RnubOR1), two EsemORs (EsemOR1 and 6), one LcapOR (LcapOR15) and one PxylOR (PxylOR3) share a common ancestor with the PR clade, although the position of LcapOR15 and PxylOR3 had low bootstrap support (<20), and is inconsistent with our previous analysis (Yuvaraj et al., 2018b). Based on the specific response of EsemOR1 to the plant volatile β-caryophyllene (Yuvaraj et al., 2017; indicated in **Figure 2**), there is currently no evidence to suggest that these ORs should be included in the PR clade.

As previously reported (Engsontia et al., 2014; Koenig et al., 2015), ORs from *P. xylostella* showed relatively large species-specific lineage expansions both within and outside the PR clade. In contrast, no major species-specific OR lineage expansions were evident among the three studied species, although a few minor expansions of 4–5 ORs could be observed (**Figure 2**). The remaining ORs from *R. nubila, E. semipurpurella*, and *L. capitella* were generally clustered basally or sister to subfamilies containing ORs from *M. sexta, E. postvittana*, and *P. xylostella*, across the tree. Several simple one-to-one orthologous relationships with bootstrap support >70% were evident between ORs in *R. nubila*, *E. semipurpurella*, and *L. capitella*: RnubOR3/LcapOR36, RnubOR15/EsemOR24, RnubOR29/EsemOR26, RnubOR31/LcapOR32, EsemOR7/Lcap OR23, and EsemOR9/LcapOR22 (all indicated in **Figure 2**).

The estimated expression levels (FPKM values) of the EsemORs and LcapORs were reported previously (Yuvaraj et al., 2017, 2018b). In terms of sex-biased expression, *L. capitella* has 7 ORs with estimated male-biased expression of which LcapOR6, 7, and 8 are located within the PR clade (**Figure 2**). Three LcapORs have female-biased FPKM values of which LcapOR3 is within the PR clade (**Table 2**). In *R. nubila*, 6 ORs have male-biased expression, and 2 ORs female-biased expression (**Table 2**). For *E. semipurpurella*, we did not have access to a female antennal transcriptome (**Supplementary Data Sheets S1**–**S3**).

**Table 2:** Chemosensory genes from *R. nubila* and *L. capitella* with estimated sex-biased expression (>3-fold difference), presented as FPKM (Fragments Per Kilobase of transcript per Million mapped reads) values.

	*Rhyacophila nubila*			*Lampronia capitella*		
		
	Chemosensory genes	Male FPKM	Female FPKM	Chemosensory genes	Male FPKM	Female FPKM
Male-biased expression	OR11	181	25.4	OR6	203	5.37
	OR17	7.14	0.59	OR7	8.23	0.06
	OR18	46.0	7.92	OR8	219	14.2
	OR20	18.5	5.85	OR32	2.59	0.86
	OR21	25.9	5.48	OR41	6.02	1.91
	OR31	3.89	0.82	OR50	76.9	0.93
				OR51	14.2	0.98
	OBP8	2.07	0.27	PBP2	15155	2841
	OBP18a	4860	753	OBP1	8179	2347
	OBP18b	3288	549	OBP8	2192	19.6
	OBP27	9.64	2.95	OBP20	153	43.5
	IR8a	70.5	12.1			
	IR75q.0.2	275	24.5			
				SNMP1b	46.9	4.36
				CSP17	20.5	6.28
Female-biased expression	OR22	1.14	4.06	OR3	1.25	53.7
	OR24	0.82	3.70	OR28	1.53	25.4
				OR29	1.64	21.6


#### Gustatory Receptors

We identified 9 GRs (6 full-length) in *R. nubila*, 3 GRs (1 full-length) in *E. semipurpurella*, and 5 GRs (2 full-length) in *L. capitella* (**Table 1** and **Supplementary Data Sheets S1**–**S3**). Among these GRs, orthologs of the three carbon dioxide receptors were identified in *L. capitella* (LcapGR1-3) based on sequence homology; two of them were found in *R. nubila* (RnubGR1 and RnubGR2), but none of them was found in *E. semipurpurella*. Two putative non-fructose sugar receptors were identified in *R. nubila* (RnubGR4 and RnubGR6) as well as in *E. semipurpurella* (EsemGR4 and EsemGR6), whereas one was found in *L. capitella* (LcapGR4). One putative fructose receptor was found in each of *R. nubila* and *L. capitella* (RnubGR9 and LcapGR9). The remaining GRs (RnubGR11-14 and EsemGR11) were regarded as putative bitter taste receptors. In general, the GRs had low FPKM values and none of them was sex-biased (**Supplementary Data Sheets S1**–**S3**). Due to the small number of GRs identified, which is expected for antennal transcriptomes, we do not report a phylogenetic analysis for this gene family.

#### Ionotropic Receptors

**FIGURE 3:**
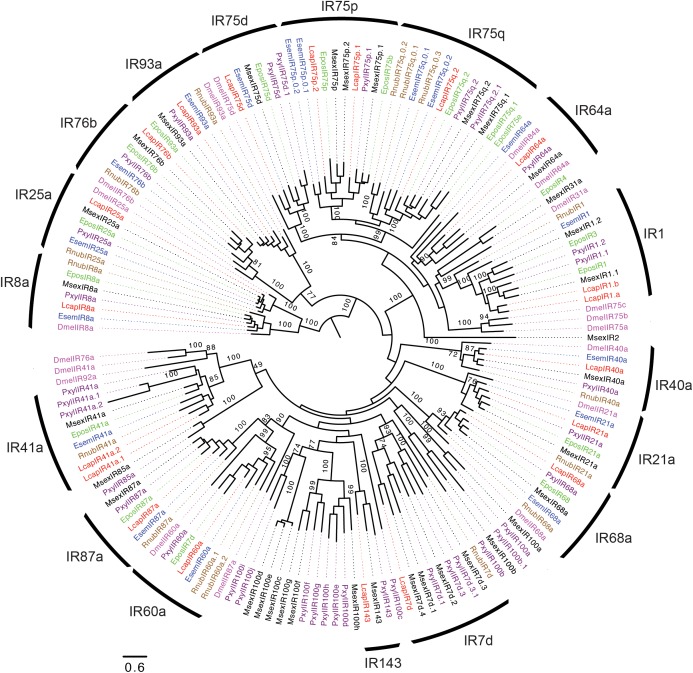
Maximum-likelihood phylogram of ionotropic receptors (IRs), rooted with the IR8a and IR25a subfamilies. Included are IRs from *R. nubila* (brown), *L. capitella* (red), *E. semipurpurella* (blue), *E. postvittana* (green), *M. sexta* (black), *P. xylostella* (purple), and conserved IRs from *Drosophila melanogaster* (Magenta). Bootstrap support values (500 replicates) are indicated when >70%. Sources of included IR sequences, along with their accession numbers (when applicable), are reported in **Supplementary Data Sheet S4**.

In total, 17 IRs were identified in *R. nubila*, 17 in *E. semipurpurella*, and 20 in *L. capitella* (**Table 1** and **Supplementary Data Sheets S1**–**S3**). The conserved antennal IRs (Croset et al., 2010) and IRs belonging to the so-called ‘Lepidoptera-specific’ IR subfamilies (Liu et al., 2018) were named based on their orthologous relationships with members in other species. Collectively in the three species, we found orthologs for the ‘Lepidoptera-specific’ receptors IR1 and IR87a, and the antennal receptors IR8a, IR21a, IR25a, IR40a, IR41a, IR60a, IR68a, IR76b, IR93a, and several members of the IR75 group, including IR75d, IR75p, and IR75q (**Figure 3**; Croset et al., 2010). The IR75p and IR75q proteins from *L. capitella* were further classified based on their phylogenetic positions within the subfamilies of IR75p.1, p.2 and q.2 proteins from other lepidopterans (no IR75q.1 ortholog was found in *L. capitella*). However, the two IR75p relatives from *E. semipurpurella* were positioned sister to the entire subfamily of IR75p.1 and p.2 proteins, and were hence named EsemIR75p.0.1 and p.0.2. Similarly, two IRs from *E. semipurpurella* and three IRs from *R. nubila*, all related to IR75q, could not be assigned to the specific subfamilies IR75q.1 or q.2. Hence, they were named EsemIR75q.0.1 EsemIR75q.0.2, and RnubIR75q.0.1-q.0.3. We found two members of IR41a in *L. capitella* (LcapIR41a.1 and LcapIR41a.2), and two members of IR60a in *R. nubila* (RnubIR60a.1 and RnubIR60a.2). Of the above-mentioned orthologs, we did not find all of them in each of the three species. Specifically, IR64a, IR75d, and putative IR75p members were identified in *E. semipurpurella* and *L. capitella*, but not in *R. nubila*. In addition, an ortholog to one of the divergent IR subfamilies of Lepidoptera, IR7d, was found in *R. nubila* and *L. capitella*, but not in *E. semipurpurella.* The occurrence of an ortholog of the ‘Lepidoptera-specific’ IR87a and a member of the IR1 group also in *R. nubila* suggest that these IRs also occur in Trichoptera. An ortholog of the IR143 group was found only in *L. capitella*.

Ten IRs from *R. nubila*, 12 IRs from *E. semipurpurella* and 12 IRs from *L. capitella* were putatively full-length, whereas the rest of them are represented as partial genes (**Supplementary Data Sheets S1**–**S3**). The putative IR co-receptors, IR8a and IR25a, were among the most highly expressed IR transcripts in the three species. IR25a was estimated to be expressed 2–5 times higher than IR8a in *R. nubila* and *L. capitella* (**Supplementary Data Sheets S1**–**S3**). However, in *E. semipurpurella* the expression of IR25a was low compared to that of IR8a. In addition, RnubIR75q.0.2 and LcapIR76b showed particularly high antennal expression in these species. RnubIR75q.0.2 and RnubIR8a showed male-biased expression (**Table 2**).

### Non-receptor Chemosensory Gene Families

#### Odorant Binding Proteins

**FIGURE 4:**
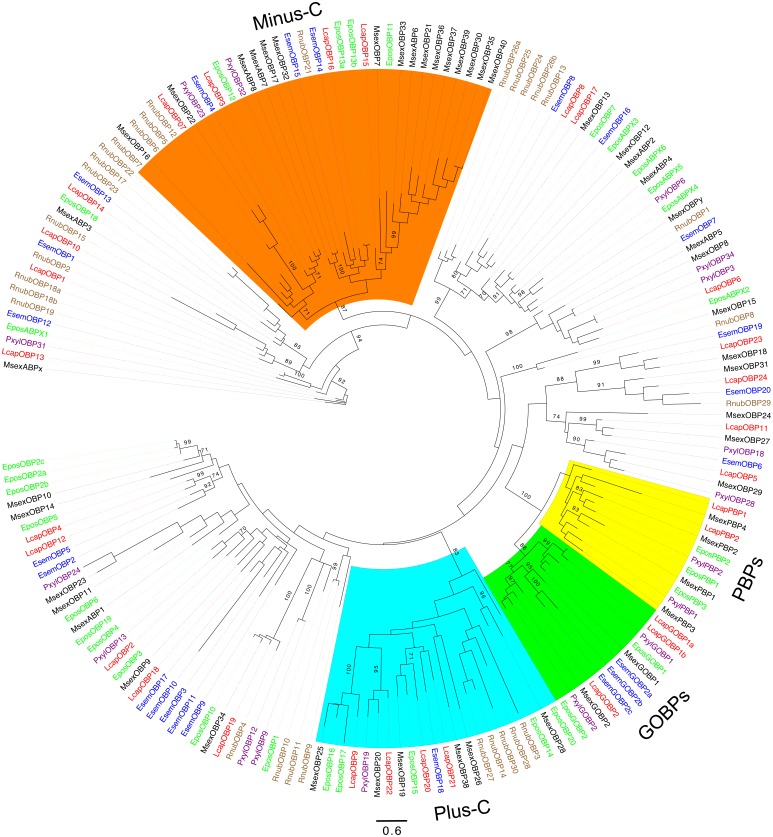
Unrooted maximum-likelihood phylogram of odorant binding proteins (OBPs). Included are sequences from *R. nubila* (brown), *L. capitella* (red), *E. semipurpurella* (blue), *E. postvittana* (green), *M. sexta* (black), and *P. xylostella* (purple). Highlighted subfamilies; pheromone binding proteins, PBPs (yellow), general odorant binding proteins, GOBPs (green), Plus-C OBPs (cyan) and Minus-C OBPs (orange). Bootstrap support values (500 replicates) are indicated on major branches when >70%. Sources of included OBP sequences, along with their accession numbers (when applicable), are reported in **Supplementary Data Sheet S4**.

We identified 30 transcripts encoding OBPs in *R. nubila* (23 full-length), 23 in *E. semipurpurella* (20 full-length) and 29 in *L. capitella* (24 full-length) (**Table 1** and **Supplementary Data Sheets S1**–**S3**). OBPs are classified into different sub-groups according to the patterns of conserved cysteine residues, and in Lepidoptera also based on phylogenetic position and putative function (**Figure 4**). Classic OBPs have six conserved cysteines, whereas the Plus-C class has 12 cysteines and one characteristic proline residue (Hekmat-Scafe et al., 2002; Sánchez-Gracia et al., 2009; Fan et al., 2011). The Minus-C class (generally) lacks two of the six conserved cysteines, i.e., those at positions two and five. We found 5 RnubOBPs, 1 EsemOBPs, and 4 LcapOBPs that belong to the Plus-C class, and 5 RnubOBPs, 3 EsemOBPs, and 4 LcapOBPs that belong to the Minus-C class (**Figure 4**).

In Lepidoptera, the PBPs (Pheromone Binding Proteins) and GOBPs (General Odorant Binding Proteins) form two monophyletic subfamilies, together sharing a common ancestor, and they appear conserved in ditrysian moths (Vogt et al., 2015). In both *E. semipurpurella* and *L. capitella*, we found three members that grouped in the GOBP clade (**Figure 4**). The three EsemGOPBs were all most closely related to members of the GOBP2 subfamily. In contrast, two of the LcapGOBPs were related to the GOBP1 clade, and one was related to the GOBP2 clade. No OBPs from *R. nubila* grouped within the GOBP clade. Also, we did not find any OBP member that could be classified as a PBP in *R. nubila* or *E. semipurpurella* (both using Type 0 pheromones), but we found two PBP members in *L. capitella* (using a Type I pheromone). LcapPBP1 fell at a position sister to the rest of the PBP clade, but with low bootstrap support. LcapPBP2 grouped together with MsexPBP4, which has been suggested to belong to the PBP-B sub-family (Vogt et al., 2015). The estimated expression levels of the OBPs were in general high. In addition, the FPKM values of a few OBPs indicated male-biased expression, i.e., RnubOBP8, 18a, 18b, 27, and LcapPBP2, OBP1, 8, and 20 (**Table 2**).

#### Chemosensory Proteins

**FIGURE 5:**
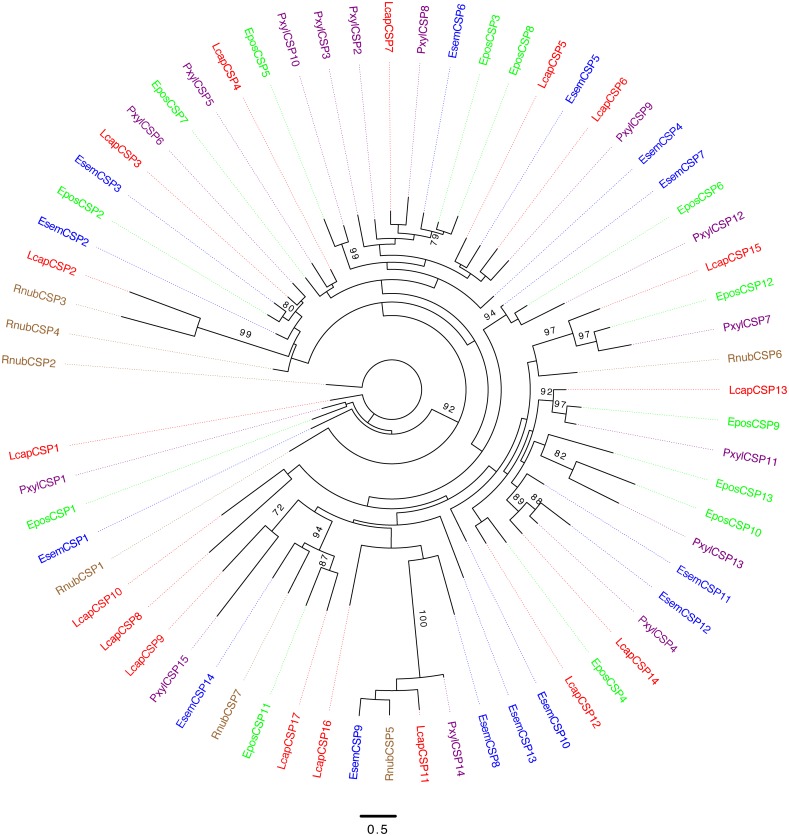
Unrooted maximum-likelihood phylogram based on protein sequences of chemosensory proteins (CSPs). Included are sequences from *R. nubila* (brown), *L. capitella* (red), *E. semipurpurella* (blue), *E. postvittana* (green), and *P. xylostella* (purple). Bootstrap support values (500 replicates) are indicated on major branches when >70%. Sources of included CSP sequences, along with their accession numbers (when applicable), are reported in **Supplementary Data Sheet S4**.

We identified seven transcripts encoding CSPs from *R. nubila* (all full-length), 14 from *E. semipurpurella* (9 full-length) and 17 from *L. capitella* (15 full-length) (**Table 1** and **Supplementary Data Sheets S1**–**S3**). Some of the CSPs were indicated as highly expressed in the three species, but none of them as abundant as the most highly expressed OBPs or PBPs (**Supplementary Data Sheets S1**–**S3**). The CSPs from all three species were scattered across the phylogenetic tree, clustering together with CSPs from the other species (**Figure 5**). One CSP had estimated male-biased expression in *L. capitella* (**Table 2**).

#### Sensory Neuron Membrane Proteins

**FIGURE 6:**
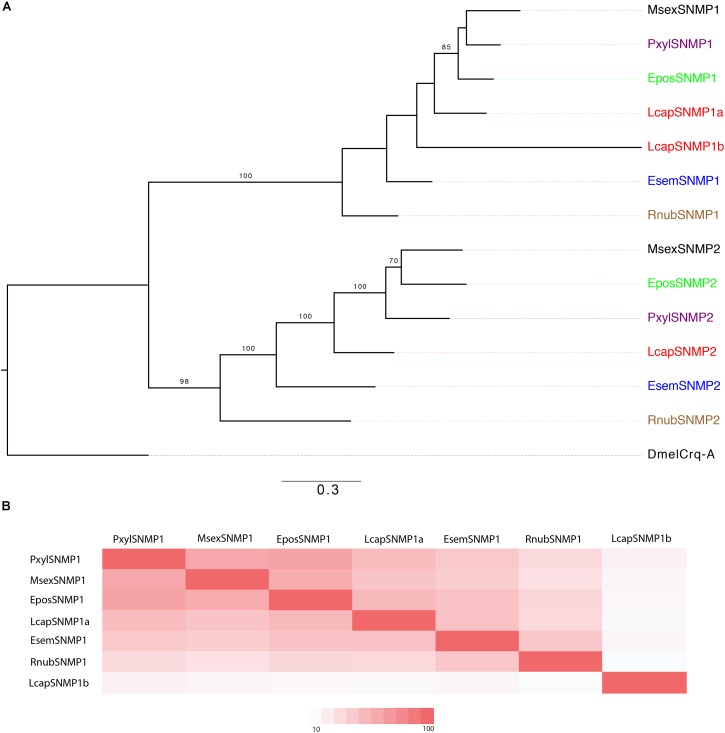
**(A)** Maximum-likelihood phylogram of sensory neuron membrane proteins (SNMPs), rooted with a non-SNMP member of the CD36 family (Croquemort, Dmelcrq-A). Included are SNMP sequences from *R. nubila* (brown), *L. capitella* (red), *E. semipurpurella* (blue), *E. postvittana* (green), *M. sexta* (black), and *P. xylostella* (purple). Bootstrap support values (500 replicates) are indicated when >70%. Sources of included SNMP sequences, along with their accession numbers (when applicable), are reported in **Supplementary Data Sheet S4**. **(B)** Comparison of amino acid sequence identity between trichopteran and lepidopteran SNMP1s that are included in the tree.

We identified one member of each of SNMP1 and SNMP2 in *R. nubila* and *E. semipurpurella*. In *L. capitella*, we found two orthologs of SNMP1 (labeled SNMP1a and SNMP1b) and one ortholog of SNMP2 (**Figure 6A** and **Table 1**). Comparing sequence identity, LcapSNMP1a appeared more conserved than LcapSNMP1b with the former sharing 50–75% identity with SNMP1 members from the other moth species included in this analysis. In contrast, LcapSNMP1b, with male-biased expression (**Table 2**), shared about 40% sequence identity with the other SNMP1 orthologs (**Figure 6B**). The shared sequence identity of SNMP2 orthologous was lower, ranging between 30 and 65% across species. All the SNMP transcripts from *R. nubila*, *E. semipurpurella*, and *L. capitella* represent full-length genes.

## Discussion

Prior to this study, the chemosensory gene families had been identified from many species that belong to more recent lineages of Lepidoptera (Ditrysia). This is the first study reporting the identification and evolutionary analyses of the chemosensory gene families from the early-diverging lineages of the Lepidoptera, as well as its sister order Trichoptera. As such, our study enhances the compendium of chemosensory genes in these taxa, providing a foundation for improved evolutionary analyses and functional characterization.

The numbers of putative OR transcripts identified in *R. nubila* and *E. semipurpurella* (37 in both species) were lower than the number (53) identified in *L. capitella*. This suggests that fewer ORs are expressed in the adult antennae of trichopterans as well as in the oldest lepidopteran lineages, as compared to more recent lepidopteran lineages and many other groups of insects (Grosse-Wilde et al., 2011; Zhan et al., 2011; Bengtsson et al., 2012; Andersson et al., 2013, 2014; Engsontia et al., 2014; Corcoran et al., 2015 Dippel et al., 2016; Walker et al., 2016). Indeed, different OR subfamilies have expanded to various degrees in different insect taxa, which possibly reflects differences in ecological specialization (Nei et al., 2008; Hansson and Stensmyr, 2011; Missbach et al., 2014; Andersson et al., 2015; Benton, 2015). However, for *E. semipurpurella* we could only analyze the male antennal transcriptome, and therefore, ORs with female-specific expression might have been missed. In addition, our BUSCO analysis indicated that the completeness of *E. semipurpurella* assembly was lower than that for *L. capitella* (86% vs. 95%), which could partly contribute to the difference in OR numbers observed between these two species. Whether the older lepidopteran lineages and trichopterans in general express fewer antennal ORs than most species of moths should be confirmed by analysis of additional species. As expected, larger numbers of ORs have been identified in the genomes of several moth species with total counts ranging from 64 to 79 (International Silkworm Genome Consortium, 2008; Zhan et al., 2011; *Heliconius* Genome Consortium, 2012; Engsontia et al., 2014; Koenig et al., 2015). The numbers of ORs encoded by the genomes of the three analyzed species are likely to exceed those reported from the antennal transcriptomes.

*Lampronia capitella* has seven ORs that group within the lepidopteran PR clade. Three of these ORs responded to Type I pheromone compounds (Yuvaraj et al., 2018b; **Figure 2**). In contrast, no ORs from *R. nubila* or *E. semipurpurella* group within the PR clade, and the functionally characterized PRs for Type 0 pheromones in *E. semipurpurella* have an independent evolutionary origin (Yuvaraj et al., 2017). However, one OR from *R. nubila* and two ORs from *E. semipurpurella* are positioned sister to the PR clade and thus appear to share a common ancestor with the PRs of species using Type I pheromones (**Figure 2**). Among these ORs, EsemOR1 responded only to the plant volatile β-caryophyllene. This result led to the hypothesis that the PRs within the PR clade might have evolved their role as sex pheromone detectors from ORs that detect plant volatiles (Yuvaraj et al., 2017). The functional studies of PRs in non-ditrysian lepidopterans suggest that receptors within the PR clade gained a novel function as pheromone detectors in association with the transition from Type 0 to Type I pheromones early in the radiation of the Lepidoptera (Yuvaraj et al., 2018b). However, within the PR clade, there are many receptors with unknown ligands (Engsontia et al., 2014; Koenig et al., 2015; Yuvaraj et al., 2017). In order to improve our understanding of the function and evolution of the receptors within the PR clade, functional studies of ORs from additional lepidopteran lineages, particularly older ones, are necessary.

Several putative one-to-one orthologous relationships were found between ORs from the three studied species (**Figure 2**), suggesting that some olfactory functions might be conserved among the older lepidopteran lineages and even with Trichoptera. In contrast, very few simple orthologous relationships were evident among the ORs in these moths and those from species in ditrysian families. Instead, the Rnub, Esem, and LcapORs were regularly positioned basally in major lepidopteran OR subfamilies. These patterns of OR relationships are consistent with the species phylogeny, and suggest a phylogenetic signal in the evolution of the OR gene family. *R. nubila* and *L. capitella* contain six and seven ORs with male-biased FPKM values, respectively, with the male-biased RnubOR21 grouping close to the Type 0 PRs in *E. semipurpurella* (**Figure 2**; Yuvaraj et al., 2017). It is possible that the male-biased RnubORs are involved in the detection of the female produced sex pheromone, but this hypothesis remains to be tested. In addition, quantitative RT-PCR should be performed to verify the sex-biased expression indicated by FPKM values in this study.

The interplay between PBPs and PRs probably facilitates pheromone detection and specificity in moths (Große-Wilde et al., 2006; Forstner et al., 2009; Leal, 2013; Sun et al., 2013). The GOBPs and PBPs form two subfamilies within a Lepidoptera-specific clade, but they had previously only been identified in ditrysian species (Hekmat-Scafe et al., 2002; Vogt et al., 2002; Pelosi et al., 2006; Vieira and Rozas, 2011; Vogt et al., 2015). We did not find any binding proteins that were related to GOBPs or PBPs in *R*. *nubila*. Also *E. semipurpurella* appears to lack antennally expressed PBPs, however, in this species we identified three GOBPs. In *L. capitella*, we identified both GOBPs and PBPs, representing the first identification of PBPs in a non-ditrysian moth. It has been suggested that PBPs and GOBPs may be mostly associated with pheromone-detecting sensilla trichodea and plant volatile-sensitive sensilla basiconica, respectively (Vogt et al., 1991; Maida et al., 2005; Forstner et al., 2009; Vogt et al., 2015; but see Vogt et al., 2002; Nardi et al., 2003). The presence or absence of PBPs in the antenna may be related to the type of pheromone compounds used. For instance, *R*. *nubila* and *E. semipurpurella* produce Type 0 pheromone compounds whereas *L. capitella* uses a Type I pheromone (Löfstedt et al., 2016). As mentioned previously, the PRs for Type 0 pheromones in *E. semipurpurella* have probably evolved from plant odor-detecting ORs, and the characterized EsemPRs also responded secondarily to plant volatiles (Yuvaraj et al., 2017). Thus, due to the structural similarity between Type 0 pheromones and common plant volatiles, it is possible that GOBPs are associated with the detection of Type 0 pheromone compounds in *E. semipurpurella*. If so, it is surprising that no GOBPs were found in *R*. *nubila*, which also uses a Type 0 pheromone. Functional characterization of OBPs from these and additional species from the older Lepidoptera is necessary to test this hypothesis. Nevertheless, the current data suggest that GOBPs are found throughout the Lepidoptera, whereas PBPs appear to be associated only with species using Type I pheromones, at least when considering antennal expression.

Most of the conserved antennal IRs that are found across insects (e.g., Croset et al., 2010; Koenig et al., 2015; Zhao et al., 2015; Dippel et al., 2016; van Schooten et al., 2016; Schoville et al., 2018) were identified in this study. However, a few of the orthologs were not found in all species, which could be due to low antennal expression of some of these IRs. In addition, we found very few IRs of the divergent class (Croset et al., 2010), which was expected because these IRs are primarily expressed in gustatory tissues (Rytz et al., 2013; Koh et al., 2014; van Schooten et al., 2016). Interestingly, we identified several IRs not previously reported outside ditrysian Lepidoptera (Koenig et al., 2015; van Schooten et al., 2016; Liu et al., 2018). Specifically, we identified the first IR143a ortholog in a non-ditrysian moth (*L. capitella*), IR7 members in both *L. capitella* and the trichopteran *R. nubila*, as well as IR87a and IR1 members in both non-ditrysian Lepidoptera and in Trichoptera. Hence, the evolutionary radiation of several IR subfamilies appears to have started prior to the split of the two sister orders Trichoptera and Lepidoptera.

In *D. melanogaster* and moths, SNMP1 is important for the responses of some pheromone receptors (Benton et al., 2007; Li et al., 2014; Pregitzer et al., 2014; Gomez-Diaz et al., 2016). The SNMPs are conserved across insects (Nichols and Vogt, 2008; Vogt et al., 2009), and we identified them also in our study species. Several species have multiple members of SNMP1 (Nichols and Vogt, 2008; Andersson et al., 2013, 2014), and *L. capitella* has two members expressed in the antennae. While the sequence of LcapSNMP1a is similar to those of SNMP1 members in other moths and in *R. nubila*, LcapSNMP1b is more divergent, also in comparison to LcapSNMP1a (**Figure 6B**). Similarly, the six putative SNMP1 members in the Hessian fly, *Mayetiola destructor* (Diptera, Cecidomyiidae), share only 29–45% sequence identity (Andersson et al., 2014, 2016). The evolutionary forces driving divergence among multiple SNMP1 members within a species remain unknown, but relaxed purifying selection following duplication events might play a role, similar to what has been proposed for OR evolution (Zhang and Löfstedt, 2013; Andersson et al., 2015; Benton, 2015; Zhang and Löfstedt, 2015). In addition, the function of multiple SNMP1 members within a species remains to be unraveled, whether olfactory or not. In the Hessian fly, the responses of MdesOR115 to minor pheromone components were not affected by co-expression of the different SNMP1 members when tested *in vitro* (Andersson et al., 2016). However, this result does not rule out an important role for any of the different SNMP1s *in vivo*.

## Conclusion

Our transcriptome analysis provides the first set of chemosensory genes from the older Lepidoptera and a species of Trichoptera, facilitating the evolutionary analysis of these gene families in these two diverse orders of Insecta. In addition to showing that several subfamilies of chemosensory genes are shared between these orders, our results suggest that the conserved PR clade of Lepidoptera and the PBPs have emerged in parallel with the evolution of Type I sex pheromones, although this hypothesis should be tested by genome analysis. Future studies should aim to characterize the function of these olfactory proteins to further our understanding of the relationship between species ecology, pheromone communication, and the evolution of olfactory proteins in relation to species diversification.

## Author Contributions

JY, MA, and CL conceived and designed the study. JY collected biological material. JY performed molecular work with assistance from D-DZ. JY and MA performed transcriptome data analysis and constructed the phylogenetic trees. JY and MA wrote the manuscript together with contributions from D-DZ and CL. All authors read and approved the final version of the manuscript.

## Conflict of Interest Statement

The authors declare that the research was conducted in the absence of any commercial or financial relationships that could be construed as a potential conflict of interest.

## Footnotes

1 http://www.usadellab.org/cms/?page=trimmomatic

2 https://busco.ezlab.org/

3 http://pfam.xfam.org/search/sequence

4 http://www.cbs.dtu.dk/services/TMHMM-2.0/

5 http://www.geneious.com

6 http://tree.bio.ed.ac.uk/software/figtree/

## Supplementary Material

TABLE S1 Oligonucleotide primer sequences of cloned (full length) and RACE-PCR-extended odorant receptor (OR) and ionotropic receptor (IR) genes.

TABLE S2 Assessment of transcriptome assembly completeness using the Benchmarking Universal Single-Copy Orthologs (BUSCOv3) tool performed against the Insecta odb9 dataset (https://busco.ezlab.org/).

DATA SHEET S1 Sequences (DNA and protein), length details, and expression levels of the chemosensory genes identified in *Rhyacophila nubila*.

DATA SHEET S2 Sequences (DNA and protein), length details, and expression levels of the chemosensory genes identified in *Eriocrania semipurpurella*.

DATA SHEET S3 Sequences (DNA and protein), length details, and expression levels of the chemosensory genes identified in *Lampronia capitella*.

DATA SHEET S4 Accession numbers and/or source references for the protein sequences included in the trees presented in **Figures 2**–**6**.
