# Supplementary material for: Antennal Transcriptome Analysis of the Chemosensory Gene Families From Trichoptera and Basal Lepidoptera
Source: Front Physiol. 2018 Sep 27;9:1365. doi: 10.3389/fphys.2018.01365 (PMC6171000; doi:10.3389/fphys.2018.01365)
Supplement: TABLE S1 — Oligonucleotide primer sequences of cloned (full length) and RACE-PCR-extended odorant receptor (OR) and ionotropic receptor (IR) genes. [file Table_1.DOCX]

**Supplementary Table 1.** Oligonucleotide primer sequences of cloned (full length) and RACE-PCR-extended odorant receptor (OR) and ionotropic receptor (IR) genes.

| **Gene** | **Forward primer (5’→ 3’)** | **Reverse primer (3’→ 5’)** |
| --- | --- | --- |
| RnubOrco | ATGAACAAATTCAAAGTCCACGGTC | CTATTTAAGCTGGACGAGCACCATGAA |
| RnubOR1 | TGGTAGCACATTTTACTACAATTATGAAAGGA | TCACTCATCAAAACTTCGAAGAATCG |
| RnubOR3 | ATGGGCGAAATTTTCGTCGATATC | TCATTTATTGTGCATGTGTTGAAGTACCG |
| RnubOR4 | ATGCTCAATATGATTTCCATTAAAAAGAAG | TTATTGCTTTGTAGAACGCAGC |
| RnubOR5 | ATGGAATTATATCAAAGCAAACTTTTATCGTTCAG | ATTGCGGGCCCTTATTTAAAGACAGC |
|  | 5’ RACE- AATGCAGCGGCTGTCGAGAGGAACGGC |  |
| RnubOR6 | ATGGTAAAAATCGAGAGCAGACG | TCATAGTTGCTTTTTGAAACTCATCAC |
| RnubOR7 | ATGTATATAGATCATCGAAAGCATAAATATGATA | TCAGTAGATCTCTTCTCGTAGAGTGTTC |
| RnubOR8 | ATGTCAATATTTCAACAAATAAAAAAAAGGAATTG | TTACTTTGCAAAGACATTATAAATAGTGTAGG |
|  | 5’ RACE- GTCCCCAGTTCCGACCAGACCTTCGGC |  |
| RnubOR9 | ATGGACGAGTCATCAAAGTTGAGATTAAC | TTATTTCGAATTGAGTTGTTGGAGTACTGC |
| RnubOR10 | ATGTTCCAGAATTTTTTGTCATTTTTTCAC | TTAGTCAATCACTATTTTTCCTTTCTTCAC |
| RnubOR11 | ATGGCAGAATTAGAAGAAAGCTTAGAGC | TTATTCTACAGCCCCTTCGTACATG |
| EsemOrco | ATGACCAAATTCAAAGTGCAAGGC | CTACTTGAGTTGCACCAACACCATG |
| EsemOR1 | ATGGTAGGAAATAAATTCAGACAATTATCCTCG | TCACTGTACCATCGTCTCGAGAG |
| EsemOR3 | ATGGCGGAGTTGTACGAGG | TTATTCCATGGGTTCATTCATCGAG |
| EsemOR4 | ATGATTTGTCAGGAAAAAATGGACAC | TCATTCTTTTTCTTCTCCGACAGAC |
| EsemOR5 | ATGGTTTACGAAGATCAATTGCACG | TTACTCTGCTCCCATCGAACTC |
| EsemOR6 | ATGTCGGAGAATATTCCGATACAGAG | TCACTCCAACGTCTGCAGC |
| LcapOrco | ATGACCAAATTCAAAGTGCAGGGCC | AGTATGGGCCCCTATTTGAGTTGCACCAG |
| LcapOR1 | ATGGAGCGGAGCAAACGAAAAGACCC | TTGCGGGCCCCTAGTTCATACTCGTAC |
| LcapOR3 | ATGAATTTTGTGCTTGAAAAATTCAAATTGGATCC | TTGCGGGCCCTTAATTAGTGCTGCTG |
| LcapOR4 | ATGGAACGTCGTACTAATCGAAGAGACCCC | TTGCGGGCCCTTAATTTAAATTCATGCTTTTCAGG |
| LcapOR5 | ATGGATCCAAAGCTGGTCTTGAGCCC | TTGCGGGCCCTTAGTTATTGTCAACTGAC |
| LcapOR6 | ATGGAAGTCGTTGCCAAACGACGAGAC | TTGCGGGCCCCTACTCCAAACTATTCAAG |
| LcapOR7 | ATGAGTTTTCTACTGCAAAAAATCAAATTGGATCC | TTGCCTCGAGTTAATTATTGTGGCTGC |
| LcapOR8 | ATGACAAGTGACGCGGTAGTTCAACTGC | TTGCGGGCCCCTACTCCAAACTATTC |
| LcapIR41a | ATGTTGACTCCGATCCAGTCATTC | TTATTTTCGTGTATTAAATAGGATATCTCCTCG |
