# Supplementary material for: Antennal Transcriptome Analysis of the Chemosensory Gene Families From Trichoptera and Basal Lepidoptera
Source: Front Physiol. 2018 Sep 27;9:1365. doi: 10.3389/fphys.2018.01365 (PMC6171000; doi:10.3389/fphys.2018.01365)
Supplement: TABLE S2 — Assessment of transcriptome assembly completeness using the Benchmarking Universal Single-Copy Orthologs (BUSCOv3) tool performed against the Insecta odb9 dataset (https://busco.ezlab.org/). [file Table_2.DOCX]

**Supplementary Table 2.** Assessment of transcriptome assembly completeness using the Benchmarking Universal Single-Copy Orthologs (BUSCOv3) tool performed against the Insecta odb9 dataset (https://busco.ezlab.org/).

|  | *R. nubila* | *E. semipurpurella* | *L. capitella* |
| --- | --- | --- | --- |
| Complete BUSCOs (C) | 1503 (91%) | 1423 (86%) | 1579 (95%) |
| Complete and single-copy BUSCOs (S) | 1142 | 1149 | 1176 |
| Complete and duplicated BUSCOs (D) | 361 | 274 | 403 |
| Fragmented BUSCOs (F) | 70 | 129 | 43 |
| Missing BUSCOs (M) | 85 | 106 | 36 |
| Total BUSCO groups searched | 1658 | 1658 | 1658 |
